# Supporting mental health and wellbeing of university and college students: A systematic review of review-level evidence of interventions

**DOI:** 10.1371/journal.pone.0266725

**Published:** 2022-07-29

**Authors:** Joanne Deborah Worsley, Andy Pennington, Rhiannon Corcoran

**Affiliations:** 1 Department of Primary Care and Mental Health, University of Liverpool, Liverpool, United Kingdom; 2 Department of Public Health and Policy, University of Liverpool, Liverpool, United Kingdom; Universita degli Studi di Milano-Bicocca, ITALY

## Abstract

**Aims:**

The review of reviews had three aims: (i) to synthesize the available evidence on interventions to improve college and university students’ mental health and wellbeing; (ii) to identify the effectiveness of interventions, and (iii) to highlight gaps in the evidence base for future study.

**Methods:**

Electronic database searches were conducted to identify reviews in English from high-income OECD countries published between 1999 and 2020. All review-level empirical studies involving post-secondary students attending colleges of further education or universities that examined interventions to improve general mental health and wellbeing were included. Articles were critically appraised using an amended version of the AMSTAR 2 tool. Evidence from the included reviews were narratively synthesized and organised by intervention types.

**Results:**

Twenty-seven reviews met the review of reviews inclusion criteria. The quality of the included reviews varied considerably. Intervention types identified included: mindfulness-based interventions, psychological interventions, psychoeducation interventions, recreation programmes, relaxation interventions, setting-based interventions, and stress management/reduction interventions. There was evidence that mindfulness-based interventions, cognitive behavioural therapy (CBT), and interventions delivered via technology were effective when compared to a passive control. Some evidence suggested that the effects of CBT-related interventions are sustained over time. Psychoeducation interventions do not appear to be as effective as other forms of intervention, with its effects not enduring over time.

**Conclusions:**

The review of reviews located a sizeable body of evidence on specific interventions such as mindfulness and cognitive-behavioural interventions. The evidence suggests that these interventions can effectively reduce common mental health difficulties in the higher education student body. Gaps and limitations in the reviews and the underlying body of evidence have been identified. These include a notable gap in the existing body of review-level evidence on setting-based interventions, acceptance and commitment training, and interventions for students attending colleges in UK settings.

## Introduction

Poor mental health of further and higher education students is a growing public policy concern [1, 2]. Recent research indicates that levels of common mental health difficulties, self-harm, and suicide are increasing among young people, especially young women [3–5]. There have been particular concerns about university students, with research and official figures suggesting that there has been an increase in the number of students experiencing mental health problems over recent years. Data on young people aged 16 to 24 years from three UK National Psychiatric Morbidity Surveys (2000, 2007, and 2014) highlighted that the prevalence of common mental health problems, suicide attempts, and self-harm was similar in students and non-students [6]. Between 2007 and 2014, however, the prevalence of common mental health problems increased in female students but not in female non-students. Although the prevalence of non-suicidal self-harm increased between 2000 and 2014 in both students and non-students, a smaller proportion of students than non-students reported suicide attempts [6]. US college students are also increasingly reporting common mental health problems and suicidality [7]. It is, therefore, important for educational institutions to offer accessible and effective interventions for their students.

Research suggests that young people’s mental health is poorer during university study than before entry. In a UK study, anxiety and depression were found to be higher at mid-course compared to one-month pre-entry into university [8]. Similarly, a UK cohort study found that levels of psychological distress increase on entering university and levels of distress did not return to pre-registration levels [9]. Other studies have also demonstrated that students’ mental health is poorer during their first year of study compared to pre-entry into university [10].

Concern around students’ mental health has prompted recent focus on mental health provision [11]. Services offered within educational institutions typically include either individual or group counselling. Although these services are well-positioned to provide mental health care, many college counselling centres across the US are under-resourced and operate at full capacity during much of the year [12]. According to an online survey of UK student counselling services, there was an increase in demand for support services over a three-year period in further education sectors [13]. Similarly, there has been an increase in the number of students seeking support from university counselling services [14]. Despite this increase, the capacity of professional services to offer 1 to 1 support to large numbers of students is limited [2]. Although requests for professional support have increased substantially [15], only a third of higher education students with mental health problems seek support from counselling services in the UK [16]. Many students do not seek help due to barriers such as stigma or lack of awareness of services [17–19]. Without formal support or intervention, there is a risk of further deterioration.

Given the increase in mental health problems among students and the surge in demand for formal support [1, 20, 21], reactive services alone cannot effectively support student mental health and wellbeing [11]. Educational institutions have recognised the need to move beyond traditional forms of support and provide alternative, more accessible interventions aimed at improving mental health and wellbeing. Such institutions have unique opportunities to identify, prevent, and treat mental health problems because they support multiple aspects of students’ lives. Although interventions exist to improve general mental health and wellbeing of students, research on the effectiveness of the various interventions has not been effectively synthesised to date. To address this, we conducted a review of review-level evidence to capture the largest body of existing research on general mental health and wellbeing interventions for college and university students. As there was a substantial body of reviews to be synthesised, the purpose of our review of review-level evidence was to summarise and synthesise this evidence and identify remaining gaps and limitations in the evidence base. This review of reviews aimed to: (i) synthesize the available evidence on interventions to improve college and university students’ mental health and wellbeing; (ii) identify the effectiveness of interventions, and (iii) highlight gaps for future study. The review of reviews explored two questions:

1. What is the current evidence on interventions to improve the general mental health and wellbeing of college and university students?
2. What does the evidence tell us about the effectiveness of current interventions and what interventions are likely to be the most effective?

## Methods

### Study identification

#### Search strategy

We conducted a search of English language peer-reviewed literature of *MEDLINE and MEDLINE In Process and other Non-Indexed Citations (via OVID)*; *PsycINFO (via EBSCOhost)*; *Social Science Citation Index (via Web of Science)*; and *CINAHL Plus (via EBSCOhost)*, from 1999 (01/01/1999) to 2020 (31/12/2020), which reflects review-level evidence of interventions before the global COVID-19 pandemic. Reference lists of all eligible reviews were hand-searched in order to identify additional relevant reviews (citation ‘snowballing’). Examples of each search strategy can be found in S1 File.

#### Inclusion and exclusion criteria

We included all review-level empirical studies (reviews of Randomised Controlled Trials [RCTs] and/or Non-Randomised Studies of Interventions [NRSIs]) involving post-secondary students attending colleges of further education or universities that examined interventions to improve general mental health and wellbeing. Both universal and indicated interventions aimed at improving mental health were included. Universal interventions are aimed at students without any pre-existing mental health problems, whilst indicated interventions are aimed at students who meet criteria for mild to moderate levels of mental health problems or have acknowledged an existing mental health problem, such as depression or anxiety. Thus, studies were included involving both general student populations and students with mental health problems. Studies were excluded if they examined interventions to address specific, pre-existing neurodevelopmental conditions (e.g., attention deficit hyperactivity disorder) or focused on non-health or wellbeing outcomes (e.g., educational performance outcomes). The search was limited to English language literature. Only peer-reviewed reviews published from year 1999 onwards from high-income countries of the Organisation for Economic Co-operation and Development (OECD) were included.

#### Screening

Titles and abstracts of publications were independently screened by two reviewers (JW and AP). Full-text copies of relevant reviews were obtained and assessed independently for inclusion by two reviewers (JW and AP). Any queries or disagreements were resolved by discussion or by recourse to a third reviewer (RC).

### Assessment of methodological quality

All reviews that met the inclusion criteria were critically appraised using an amended version of the AMSTAR 2 tool [22]. The tool was amended to make it sensitive enough to differentiate between the various methodological standards of this particular body of evidence (see S2 File). The reviews were quality assessed independently by two reviewers. Based on the results of the critical appraisal, reviews were then categorised as: (i) higher methodological quality (score 10 or above); (ii) moderate methodological quality (score 6 to 9); or (iii) lower methodological quality (score 0 to 5). This is a rating/categorisation of relative methodological quality across this body of evidence.

### Data extraction and synthesis

The following data was extracted by the first author and checked for accuracy by the second author: aims, primary study design, setting/country, type of intervention, comparator (if any), population, outcomes reported, main findings in relation to the review questions, limitations, and conclusions specified by authors. Key findings from the reviews were tabulated and narratively synthesised [23]. Findings were grouped by intervention category, with evidence from higher methodological quality reviews reported first and in greater detail [following 24, 25].

## Results

**Fig 1:**
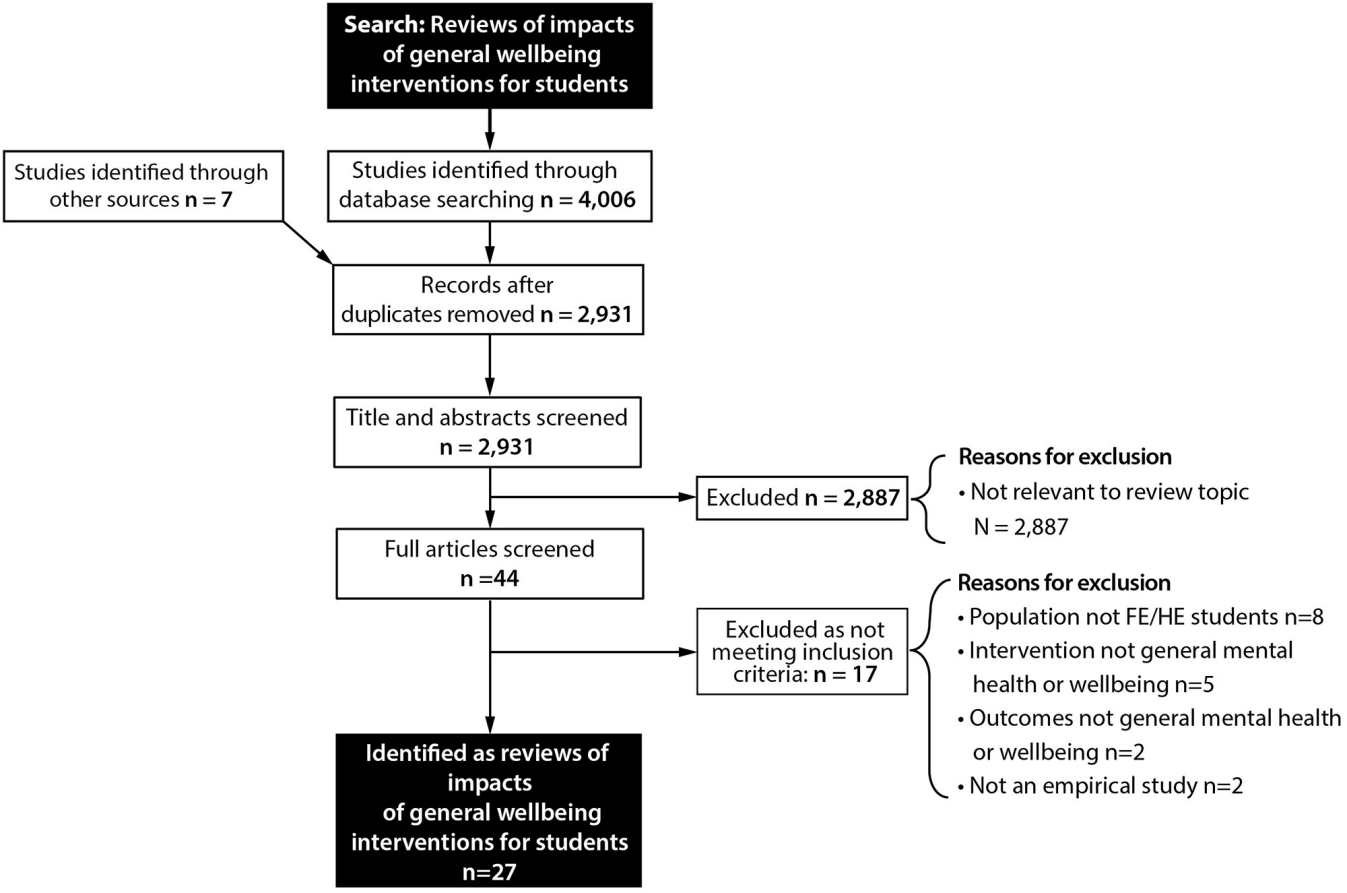
PRISMA flow chart of the progression of studies through the review.

The search generated 4,006 records. Title and abstract screening resulted in 44 articles that met the study inclusion criteria. Full-text screening resulted in the inclusion of 27 reviews. Seventeen reviews were excluded as not meeting inclusion criteria (see S3 File). A summary of our study selection process is presented in the Preferred Reporting Items for Systematic Reviews and Meta-Analyses (PRISMA) Flow Diagram (Fig 1).

### Characteristics of the included reviews

The characteristics of included reviews are summarised within Table 1. Information on setting (country) should be provided within Table 1; however, very few reviews specified the country in which interventions took place.

**Table 1:** Characteristics of the included reviews.

**Higher methodological quality**				
**Authors, date**	**Population**	**Number of included studies**	**Intervention**	**Key outcome measure**
Amanvermez et al. (2020)	College students (undergraduate or graduate)	54	Stress management interventions	Anxiety
				Depression
				Psychological distress/stress
Breedvelt et al. (2019)	Tertiary education students (university, college or other postsecondary higher education)	24	Meditation, yoga, and mindfulness	Anxiety
				Depression
				Stress
Davies et al. (2014)	Higher education students (undergraduate and postgraduate students)	17	Computer-delivered and web-based interventions	Anxiety
				Depression
				Psychological distress
				Stress
Dawson et al. (2020)	University students	51	Mindfulness-based interventions	Anxiety
				Depression
				Mindfulness
				Rumination
				Wellbeing
Guo et al. (2020)	College students	14	Exercise interventions	Depression
Halladay et al. (2019)	Post-secondary students including undergraduate, graduate, college and health professional students	49	Mindfulness-based interventions	Anxiety
				Depression
				Perceived stress
Harrer et al. (2018)	Tertiary education students (studying at university, college, or comparable post-secondary higher education)	48	Interventions delivered via technology (a specific delivery method)	Anxiety
				Depression
				Stress
				Wellbeing
Huang et al. (2018)	University or college students	51	Interventions for common mental health problems such as cognitive-behavioural interventions, mindfulness-based interventions, art, exercise, and peer support.	Anxiety
				Depression
Ma et al. (2019)	University students	25	Mindfulness-based interventions	Depressive symptoms
Winzer et al. (2018)	Students in university settings	26	Mental health promoting and mental ill health preventing interventions including CBT, mind-body interventions, and psychoeducation.	Positive mental health
				Mental ill health
**Moderate methodological quality**				
**Authors, date**	**Population**	**Number of included studies**	**Intervention**	**Key outcome measure**
Bamber and Morpeth (2019)	College students including undergraduate and postgraduate students	25	Mindfulness-based interventions (MBIs)	Anxiety
Conley et al. (2015)	Higher education students (students receiving postsecondary education in 2- or 4-year colleges and universities, trade and vocational schools, or various graduate and professional programs such as medical or law school).	90	Universal mental health prevention programs: psychoeducational interventions, cognitive-behavioral interventions, relaxation interventions, mindfulness interventions, meditation, social skills and other (e.g., psychodrama, behavioural contracting)	Anxiety
				Depression
				Stress
				General psychological distress
Conley et al. (2016)	Higher education students (college, graduate, professional)	41	Technology-delivered interventions such as cognitive behavioural interventions, mindfulness interventions, psychoeducational interventions, social skills interventions, relaxation interventions, online support group interventions, and other interventions (such as concreteness training intervention, an emotion perception training intervention, and an interactive gaming intervention).	Anxiety
				Depression
				Stress
				General psychological distress
				Health
Conley et al. (2017)	Higher education students (college, university, graduate, postgraduate and professional students)	60	Cognitive-behavioral interventions, relaxation interventions, social skills training, general behavioural interventions, social support interventions, mindfulness interventions, psychoeducational interventions, acceptance and commitment therapy interventions, interpersonal psychotherapy programs, other interventions (resilience training intervention and forgiveness training intervention).	Anxiety
				Depression
				General psychological distress
Cuijpers et al. (2016)	College students	15	Psychological therapy	Depression
Farrer et al. (2013)	Students attending a tertiary institution such as university, college, or a technical and further education (TAFE) institution	28	Technology-based interventions	Anxiety
				Depression
				Stress
Fenton et al. (2018)	Students attending postsecondary institutions	21	Recreation programs such as relaxation (mindfulness or meditation), stress management (yoga or Tai Chi), exercise (pilates), and relationships (animal therapy and expressive writing)	Anxiety
				Depression
				Stress
				Mood
Fernandez et al. (2016)	University students and staff	19	Structural and/or organizational strategies to promote mental health	Global measures of mental wellbeing, mental health, wellness or mental health related quality of life. Condition-specific outcome measures (such as depression or anxiety)
Lattie et al. (2019)	College students	89	Digital mental health interventions	Anxiety
				Depression
				Psychological wellbeing
Rith-Najarian et al. (2019)	University students	62	Prevention programmes	Anxiety
				Depression
				Stress
**Lower methodological quality**				
**Authors, date**	**Population**	**Number of included studies**	**Intervention**	**Key outcome measure**
Bamber and Schneider (2016)	College students including graduates and undergraduates.	57	Mindfulness-based interventions (MBIs)	Anxiety
				Stress
				Mindfulness
Conley (2013)	Higher education students (students receiving postsecondary education in 2- or 4-year colleges and universities, trade and vocational schools, or various graduate and professional programs such as medical or law school).	74	Universal mental health promotion and prevention programs: psychoeducational interventions, cognitive-behavioral interventions, relaxation interventions, mindfulness interventions, meditation, and others (e.g., psychodrama, behavioural contracting, expressive writing and social skills)	Emotional distress (depression, anxiety, stress, general psychological distress or wellbeing)
Howell and Passmore (2018)	University students	5	Acceptance and Commitment Training, a positive psychological intervention	Anxiety
				Depression
				Stress
				Wellbeing
Miller and Chung (2009)	College students	4	Cognitive therapy and education intervention	Depression
Reavley and Jorm (2010)	Higher education student population	Not reported	Prevention and early interventions (e.g., cognitive behavioural interventions, online support group interventions, educational/personalized feedback interventions, social marketing interventions)	Anxiety
				Depression
Regehr (2013)	Undergraduate, graduate, and professional students	24	Cognitive, behavioural and/or mindfulness-based techniques	Anxiety
				Depression
				Psychological stress
Yusufov et al. (2019)	Undergraduate and graduate students	43	Stress reduction interventions (including CBT, coping skills training, MBSR, relaxation training, psychoeducation, and social support)	Anxiety
				Perceived stress

### Overview of quality of included reviews

As shown in Table 1, the methodological quality of the reviews varied. Using the AMSTAR 2 quality assessment tool, eleven reviews were categorised as higher methodological quality, ten reviews were categorised as moderate methodological quality, and seven reviews were categorised as lower methodological quality.

### Findings of included reviews

#### 1. Mindfulness-based interventions

A systematic review and meta-analysis of RCTs (rated as higher methodological quality) of different interventions for common mental health problems in 3396 university and college students found that MBIs were effective in reducing both depression and generalized anxiety disorder in the short term but were not durable [26]. In their meta-analysis, the authors found evidence that MBIs led to statistically significant reductions in depression (pooled effect size: -0.52, 95% CI: -0.88 to -0.16). Art, exercise and peer support interventions (-0.76, 95% CI: -1.19 to -0.32), and cognitive-behavioural related interventions (-0.59, 95% CI: -0.72 to -0.45) led, however, to greater reductions. They found no evidence that the effects of MBIs on depression were sustained over time. They also found evidence that MBIs significantly reduced anxiety (-0.49, 95% CI: -0.84 to -0.15) but, again, other interventions such as peer support and music (-0.84, 95% CI: -1.19 to -0.49) and CBT related interventions (-0.39, 95% CI: -0.55 to -0.22) led to greater reductions.

Another systematic review and meta-analysis of RCTs, which was graded as higher quality, examined the effectiveness of MBIs for mental health outcomes in 4211 post-secondary students [27]. Halladay and colleagues found evidence that MBIs significantly reduced symptoms of depression (Standardised Mean Difference [SMD] -0.49, 95% CI: -0.68 to -0.30), anxiety (SMD -0.53, 95% CI: -0.78 to -0.29), and perceived stress (SMD -0.39, 95% CI: -0.50 to -0.27) when compared to a passive control group (receiving no intervention/on waiting list). There was, however, no significant difference between the MBI intervention group in levels of depression, anxiety or perceived stress when compared to an active control group receiving health education, relaxation, physical activity, or other approaches including CBT.

Halladay et al. [27] also analysed the impacts of different lengths of intervention. They found that there was no significant difference in effects, for depressive symptoms, anxiety and stress, between brief and longer interventions. They also analysed the impact of traditional compared to adapted interventions (i.e., Mindfulness-Based Stress Reduction [MBSR] versus Mindfulness-Based Cognitive Therapy [MBCT] versus other or adapted MBIs), and found that MBCT (SMD: -1.21, 95% CI: -1.76 to -0.66) was more effective than both MBSR (SMD = -0.44, 95% CI: -0.72 to -0.16, p = 0.01) and other MBIs (SMD = -0.29, 95% CI: -0.45 to -0.12, p<0.01). When compared to no intervention, MBCT was found to be the most effective type of MBI.

Studies examining whether effects were sustained over time (follow-up studies) were split by type of intervention. Halladay et al. [27] found that MBCT interventions demonstrated sustained reductions in depression one month after (post-) intervention in two studies with a total of 64 participants (Mean Difference [MD] on the Beck Depression Inventory -5.06, 95% CI: -6.52 to -3.59). Other MBIs did not demonstrate sustained reductions in depression at one month or 2–3 months post-intervention in three studies (with a total of 374 participants), although reductions in depression were found at 4–5 months post-intervention in two studies (with a total of 191 participants; SMD -0.43, 95% CI: -0.72 to -0.14). MBCT interventions also demonstrated sustained reductions in anxiety symptoms at both 1-month in two studies (with a total of 66 participants; MD on Beck Anxiety Inventory [BAI] -7.12, 95% CI: -8.23 to -5.97) and 6 months in two studies post-intervention (a total of 65 participants; MD on BAI -5.95, 95% CI: -10.78 to -1.13). Other MBIs demonstrated significant reductions 1-month post-intervention in one study using a different measure (with a total of 33 participants; MD Hamilton Anxiety Scale -9.50, CI: -17.27 to -1.73).

A systematic review and meta-analysis of RCTs (rated as higher methodological quality) of MBIs for mental and physical health in university students found that MBIs were effective in reducing distress, depression and state anxiety when compared to passive controls [28]. In their meta-analysis, the authors found evidence that MBIs led to significantly significant reductions in distress (SMD -0.47, 95% CI: -0.60 to -0.34), depression (SMD -0.40, 95% CI -0.57 to -0.24), and state anxiety (MD -3.18, 95% CI -5.51 to -0.85) when compared to a passive control (receiving no intervention/waiting list). MBIs led to improvements in wellbeing (SMD 0.35, 95% CI 0.21 to 0.50) when compared to a passive control. Effects of MBIs lasted beyond three months for distress (SMD -0.32, 95% CI -0.50 to -0.13). When compared with active control groups, MBIs significantly reduced distress (SMD -0.37, 95% CI -0.56 to -0.18) and state anxiety (MD -5.95, 95% CI -9.49 to -2.41), but not depression (SMD -0.19, 95% CI -0.43 to 0.05) and wellbeing (SMD -0.08, 95% CI -0.43 to 0.27).

Ma and colleagues conducted a meta-analytic review of RCTs (rated as higher methodological quality) of MBIs [29]. They found that MBIs were effective in reducing depressive symptoms in university students (effect size: 0.52, 95% CI 0.39 to 0.65). The authors found evidence that universal MBIs (effect size: 0.41, 95% CI 0.28 to 0.55), selective MBIs (effect size: 0.44, 95% CI 0.18 to 0.70), and indicated MBIs (effect size: 0.88, 95% CI 0.64 to 1.11) led to significant reductions in depressive symptoms.

Bamber and Morpeth’s [30] review, graded as moderate quality, included a meta-analysis of evidence on the effects of MBIs on anxiety in 1492 college students. A number of primary study designs were included: studies with two-group comparisons (e.g., MBI versus control) and studies with pre-test and post-test analysis of MBI (one-group MBI). They found MBIs significantly reduced anxiety, compared to no-treatment controls (ES 0.56, 95% CI: 0.42 to 0.70, p<0.001). MBI groups’ pre and post intervention comparisons showed large significant reductions in anxiety. There was, however, a small but significant reduction in control group anxiety pre/post comparisons. They also found that higher numbers of sessions (number not specified) increased the effects of MBIs (p = 0.01), with more sessions leading to greater reductions in anxiety.

Fenton et al. [31] conducted a moderate quality systematic review of evidence on the impacts of different recreation programmes, including MBIs, on mental health outcomes in post-secondary students in North America. Randomised controlled trials, non-randomised with control, and non-randomised no control studies were all included. They found that mindfulness interventions reduced depression, anxiety, stress, and negative mood.

Conley et al. [32] conducted a moderate quality review and meta-analysis of evidence on the impact of universal mental health prevention programmes including MBIs for higher education students. The review included two study designs: quasi-experimental and random designs. They found that skill-training programmes with supervised practice were significantly more effective than both skill-training programmes without supervised practice and psychoeducation in reducing depression, anxiety, stress, and general psychological distress. Conley and colleagues found that relaxation interventions demonstrated the most overall benefit in terms of effectiveness, followed by mindfulness interventions and cognitive-behavioural interventions that did not differ from each other.

Regehr et al. [33] conducted a review and meta-analysis (rated as lower methodological quality) of evidence on the effectiveness of preventative interventions in reducing mental health outcomes in 1431 university students, including randomised and parallel cohort designs. Regehr and colleagues found that mindfulness-based interventions focussing on stress reduction significantly reduced symptoms of anxiety and depression. In their meta-analysis, mindfulness-based interventions were assessed for their impact on anxiety. They found that mindfulness-based interventions led to significant improvements, compared to control groups (SMD -0.73, 95% CI: -1.00 to -0.45).

Conley et al. [34] reviewed evidence on the effectiveness of 83 (controlled) universal promotion and prevention interventions (rated as lower methodological quality). These authors explored whether skill-orientated interventions were more effective with or without supervised skills practice. The authors also examined the effectiveness of different strategies employed in skill-oriented interventions such as cognitive-behavioural interventions, mindfulness interventions, relaxation interventions, and meditation in quasi-experimental and random designs. They found that skill-oriented interventions were more effective with supervised practice, and that supervised skills practice interventions reduced depression, anxiety, and stress. They found mindfulness interventions to be the most effective form among the skill-oriented programmes containing supervised practice. Mindfulness interventions were significantly more effective in comparison to other interventions (the proportion of all significant post-intervention outcomes combined was 78.8% for mindfulness, in comparison to psychoeducation [12.5%], cognitive behavioural [43.4%], relaxation [27.1%], meditation [13%], and other interventions [21.9%]).

Bamber and Schneider [35] explored the effects of MBIs such as Mindfulness Based Stress Reduction (MBSR) and Mindfulness Meditation (MM) on mental health outcomes including anxiety and stress in college students (rated as lower methodological quality). Both MBSR and MM were found to significantly reduce symptoms of anxiety and stress.

#### 2. Psychological interventions (e.g., cognitive-behavioural interventions)

Huang et al. [26] conducted a systematic review and meta-analysis of RCT evidence (rated as higher methodological quality) on the effectiveness of interventions for common mental health difficulties in 3396 university and college students. They found that cognitive behavioural therapy (CBT) had significant positive effects on depression and generalized anxiety disorder. Meta-analysis results showed that cognitive-behavioural-related interventions led to greater reductions in depression (-0.59, 95% CI: -0.72 to -0.45) than mindfulness-based interventions (-0.52, 95% CI: -0.88 to -0.16) and attention/perception modification (-0.46, 95% CI: -1.06 to 0.13). Other interventions (art, exercise, and peer support) led to a greater reduction in depression (-0.76, 95% CI: -1.19 to -0.32). The follow-up (pooled) effect size of cognitive-behavioural related interventions (-0.75, 95% CI: -0.95 to -0.54) had a greater significant effect (the follow-up ranged from 2 weeks to 7 months post intervention).

CBT related interventions were associated with significant (pooled) reductions in anxiety (-0.39, 95% CI: -0.55 to -0.22). The pooled effect of other interventions (peer support and music; -0.84, 95% CI: -1.19 to -0.49) and mindfulness (-0.49, 95% CI: -0.84 to -0.15) for generalised anxiety disorder were associated with greater reductions in anxiety compared to CBT.

Winzer et al. [36] conducted a systematic review and meta-analysis (rated as higher methodological quality) to assess whether the effects of mental health promotion and mental ill-health prevention interventions were sustained over time. They found that CBT-related interventions led to significant (pooled) effects for 3–6 month and 13–18 month follow-ups in sub-group analyses for combined mental ill-health outcomes (-0.40, 95% CI-0.64 to 0.16; -0.30, 95% CI: -0.51 to 0.08, respectively). They also analysed impacts on combined positive mental health and academic performance at 3–6 months, and found that the interventions had significant effects (pooled effect size: 0.52, 95% CI: 0.06 to 0.98).

Cuijpers et al. [37] carried out a meta-analysis of evidence (rated as moderate methodological quality) that examined the effectiveness of different forms of psychological treatment, such as CBT and behavioural activation therapy (BAT), for addressing symptoms of depression in 997 college students. The review found a large overall (pooled) effect of the therapies versus controls (g = 0.89, 95% CI: 0.66 to 1.11). It also found that individual therapy was significantly more effective than group therapy (p = 0.003) but that type of treatment (CBT, BAT, or other) was not significantly associated with the size of effect.

In their review and meta-analysis (rated as moderate methodological quality) of the impact of universal mental health prevention programmes for higher education students, Conley et al. [32] found that skill-training programmes with supervised practice such as cognitive-behavioural interventions, mindfulness interventions, relaxation interventions, and meditation significantly reduced depression, anxiety, stress, and general psychological distress. Programmes without supervised practice were significantly less effective. Comparing the effectiveness of different interventions overall, they also found that relaxation interventions were the most effective (mean effect size: 0.55, 95% CI: 0.41 to 0.68), followed by CBT interventions (0.49, CI: 0.40 to 0.58), MBIs (0.34, CI: 0.19 to 0.49), meditation (0.25, CI: 0.02 to 0.53), and then psychoeducational interventions (0.13: CI: 0.06 to 0.21).

In their review and meta-analysis of evidence (rated as lower methodological quality) on the effectiveness of preventative interventions in reducing mental health outcomes in university students, Regehr et al. [33] found that cognitive and behavioural interventions focusing on stress reduction significantly reduced symptoms of anxiety and depression. In their meta-analysis, cognitive-behavioural interventions were assessed for their impact on anxiety. They found that cognitive-behavioural interventions (SDM -0.77, 95% CI: -0.97 to -0.57) led to significant improvement, compared to control groups.

Howell and Passmore [38] conducted a review and (‘initial’) meta-analysis (rated as lower methodological quality) on the impacts of ACT interventions for university student wellbeing (N = 585), including randomized controlled experimental designs. Their meta-analysis showed a small significant (pooled) effect on wellbeing (d = 0.29, 95% CI: 0.11 to 0.47, p = 0.008) when assessed with the Wellbeing Manifestations Measure Scale. ACT interventions were also found to reduce depression, anxiety, and stress.

Conley et al. [34] examined the effectiveness of different strategies employed in skill-oriented interventions such as cognitive-behavioural interventions, mindfulness interventions, relaxation interventions, and meditation (rated as lower methodological quality). Conley and colleagues found that interventions with supervised skills practice reduced depression, anxiety, and stress. Mindfulness interventions were found to be the most effective (78.8%) form of intervention among the skill-oriented programmes containing supervised practice, followed by cognitive-behavioural interventions (55.8%) which performed significantly better than relaxation (28.9%, OR = 3.11, p<0.01) and meditation (19.4%, OR = 5.26, p<0.001) interventions.

One review graded as lower quality reviewed evidence on the prevention and early intervention for mental health problems in higher education students found that CBT approaches are effective for prevention and early intervention [39]. The authors also reported that these approaches are effective for at least some months following the CBT intervention. The authors did not report the primary study designs they included.

In a literature review of studies of depression and treatment outcomes among US college students, graded as lower quality, brief individual cognitive therapy was found to be effective at reducing mild to moderate depressive symptoms [40]. This finding was based on only one RCT, however.

#### 3. Psychoeducational interventions

In their review of RCTs (graded as higher methodological quality), Winzer et al. [36] explored whether the effects of mental health interventions (e.g., psychoeducational interventions) for students in higher education were sustainable over time. They did not find significant (pooled) effects on combined mental ill health outcomes at 3–6 months, 7–12 months, or 13–18 month follow-ups. They reported no superior effect of psychoeducational intervention. The 3–6 month and 13–18 month follow-up were, however, both only based on one study.

When Conley et al. [32] reviewed evidence on the impact of universal prevention programmes for higher education students, they found that skill-training programmes with supervised practice (0.45, CI: 0.39 to 0.52) were significantly more effective than both psychoeducation (information only) interventions (0.13, CI: 0.06 to 0.21) and skill-training programmes without supervised practice (0.11, CI: -0.01 to 0.22) in reducing depression, anxiety, stress, and general psychological distress (rated as moderate methodological quality). Psychoeducational interventions yielded significant effects for several mental health related outcomes including anxiety, stress, and general psychological distress (ESs>0.13). However, these interventions did not yield significant effects for depression, social and emotional skills, or interpersonal relationships. Psychoeducational interventions were found to be less effective than relaxation interventions, cognitive-behavioural interventions, mindfulness interventions, and meditation. Although interventions with supervised skills practice produced a significant positive effect averaged across all types of outcomes at follow-up (0.28, CI: 0.16 to 0.40), psychoeducational interventions did not.

In their 2013 review (graded as lower methodological quality), Conley et al. [34] explored whether skill-oriented interventions that included supervised skills were more effective than psychoeducational programmes. They found that psychoeducational programmes were not as effective as preventive interventions for higher education students.

#### 3a. Educational/personalised feedback interventions

In their review (rated as lower methodological quality) of prevention and early intervention for mental health issues in higher education students, Reavely and Jorm [39] reported mixed findings on the effectiveness of educational/personalised feedback interventions.

Miller and Chung [40] explored treatment for depression and found that an intervention using personalised mailed feedback was effective at reducing symptoms of depression (rated as lower methodological quality). This finding was only based on one study, however.

#### 4. Recreation programmes

In their review of RCTs (rated as higher methodological quality) on the effectiveness of interventions for common mental health difficulties, Huang et al. [26] found that recreational interventions including exercise, art and peer support were effective treatments for depression and anxiety. Although both CBT and MBIs were found to be effective, other interventions (i.e., art, exercise, and peer support) showed larger effects for both depression and generalized anxiety disorder.

When exploring the combined effects of yoga, meditation, and mindfulness on depression, anxiety, and stress in 1373 tertiary education students, Breedvelt et al. [41] found moderate positive effects for yoga, meditation, and mindfulness on symptoms of depression, anxiety, and stress (rated as higher methodological quality). They found no significant differences in subgroup analysis when they compared the effectiveness of yoga, mindfulness meditation, and MBSR. A small number of the included studies (N = 6) provided long-term follow-up data which ranged from 1 to 24 months. The (pooled) effect at follow-up was found to be small to medium (g = 0.39, 95% CI: 0.17 to 0.61).

A network of meta-analysis of RCTs (rated as higher methodological quality) of exercise interventions for depression in 2010 college students found that exercise interventions were effective in reducing depression [42]. When compared with usual care, Tai Chi (SMD = -11, 95% CI -16 to -6), yoga (SMD = -9.1, 95% CI -14 to -4), dance (SMD = -5.5, 95% CI -11 to -0.39) and running (-6, 95% CI -10 to -1.6) interventions were effective in reducing depressive symptoms. The authors found Tai Chi to be the most effective exercise intervention followed by yoga.

Fenton et al. [31] reviewed evidence on the impacts of recreation programmes such as mindfulness, meditation, Tai Chi, yoga, exercise, and animal therapy on mental health outcomes in post-secondary students in North America (rated as moderate methodological quality). They included a number of different primary study designs: non-randomised with control, non-randomised no control, and RCTs. They found that mindfulness, yoga, meditation, exercise, and animal therapy all reduced depression, anxiety, stress, and negative mood.

The review of evidence (rated as moderate methodological quality) on the impact of universal mental health prevention programmes by Conley et al. [32] found that meditation interventions were more effective than psychoeducational interventions but less effective than relaxation, cognitive-behavioural and mindfulness interventions.

The review (rated as lower methodological quality) by Conley et al. [34] also examined the relative effectiveness of different approaches used in skill-oriented interventions, including cognitive-behavioural, mindfulness, relaxation, and meditation. They reported that mindfulness interventions were more effective than cognitive-behavioural interventions, relaxation interventions, and meditation; and found that cognitive-behavioural interventions were more effective than both meditation and relaxation interventions which did not differ significantly from each other.

#### 5. Relaxation interventions

In their review of universal mental health prevention programmes for higher education students (rated as moderate methodological quality), Conley et al. [32] found relaxation interventions to be the most effective. In contrast, Conley et al [34] examined the relative effectiveness of different strategies used in skill-oriented interventions including cognitive-behavioural, mindfulness, relaxation and meditation, and found that mindfulness interventions and cognitive-behavioural interventions were more effective than relaxation interventions, and that meditation and relaxation interventions did not differ significantly from each other (rated as lower methodological quality).

#### 6. Setting-based interventions

Fernandez et al. [43] conducted a systematic review of evidence (rated as moderate methodological quality) on the mental wellbeing impacts of setting-based interventions for university students. They included experimental (e.g., RCT) and observational (e.g., controlled trial without randomisation, pre-post/before and after, and time series) study designs. Academic-based interventions, to enhance learning and teaching, were found to significantly improve mental wellbeing.

#### 7. Stress management/reduction interventions

A systematic review and meta-analysis (rated as higher methodological quality) of stress management interventions for college students found that stress reduction interventions were effective in reducing distress [44]. In their meta-analysis, the authors found evidence that stress management interventions were effective in reducing stress (g = 0.61, 95% CI 0.30 to 0.93), anxiety (g = 0.52, 95% CI 0.25 to 0.78), and depression (g = 0.46, 95% CI 0.16 to 0.77) for students with high stress levels. The authors found evidence that the effects of stress management interventions were sustained over time. The effect of stress management programmes for students with high stress levels remained up to the 12-month follow-up (g = 0.40, 95% CI 0.21 to 0.60). Stress management interventions were also found to be effective in reducing depression (g = 0.36, 95% CI 0.21 to 0.51), anxiety (g = 0.52, 95% CI 0.36 to 0.68), and stress (g = 0.58, 95% CI 0.44 to 0.73) in an unselected college student population.

Yusufov et al. [45] conducted a meta-analysis (rated as lower methodological quality) of evidence on the impacts of stress reduction interventions. In their meta-analysis of stress reduction interventions, the authors found that stress reduction interventions were effective in reducing anxiety and stress.

#### Interventions delivered via technology

Different categories of interventions (e.g., CBT) can be delivered through different means. Harrer et al. [46] systematically reviewed and performed a meta-analysis of evidence (rated as higher methodological quality) on the impacts of internet interventions on symptoms of common mental health problems, wellbeing and functional outcomes among university students. Small effects from internet interventions were found on depression (*g* = 0.18, 95% CI: 0.08 to 0.27), anxiety (*g* = 0.27, 95% CI: 0.13 to 0.40), and stress (*g* = 0.20, 95% CI: 0.02 to 0.38). There were, however, no significant effects on wellbeing. The effects were higher for interventions that were based on CBT principles.

Similarly, Davies et al. [47] reviewed evidence on the effectiveness of computer-delivered and web-based interventions in improving depression, anxiety, and psychological wellbeing in 1795 higher education students (rated as higher methodological quality). When compared to an inactive control group (receiving no-treatment or on a waiting list), sensitivity meta-analyses showed that interventions significantly improved anxiety (Pooled SMD −0.56; 95% CI: −0.77 to −0.35, *p*<0.001), depression (SMD −0.43; 95% CI: −0.63 to −0.22, *p*<0.001), and stress (SMD −0.73; 95% CI: −1.27 to −0.19, *p* = 0.008). The sensitivity analyses showed no significant effects for anxiety or depression, however, when compared to the active control group (in which participants received materials designed to mimic the time and attention received in the intervention group). Sensitivity analyses also showed no significant difference between the computer and web-based intervention for anxiety or depression when compared to comparison interventions that included a face-to-face version of the intervention, a web-based stress management intervention, another computer-based CBT program, and an online support group.

Lattie et al. [48] conducted a systematic review of evidence (rated as moderate methodological quality) on the effectiveness of digital mental health interventions on mental health outcomes in college students. All study designs were included. They found that digital mental health interventions can be effective for improving depression, anxiety, and psychological wellbeing among college students.

Conley et al. [49] conducted a meta-analytic review of evidence on the impact of universal and indicated technology-delivered interventions (TDIs) targeting mental health outcomes in 4763 higher education students, including randomized and quasi-experimental study designs (rated as moderate methodological quality). Universal interventions are aimed at students without any pre-existing mental health problems whereas indicated interventions are aimed at students who meet criteria for mild to moderate levels of mental health problems or have acknowledged an existing mental health problem such as depression or anxiety. They found that both universal and indicated TDIs were significantly effective in reducing symptoms of depression, anxiety, and stress. Indicated interventions produced higher overall (mean) improvements (0.37, CI: 0.27 to 0.47, p<0.001) than universal interventions (0.19, CI: 0.11 to 0.28, p<0.001). Both universal (0.21, CI: 0.11 to 0.31, p<0.001) and indicated (0.39, CI: 0.29 to 0.50, p<0.001) skill-training interventions led to significant improvements. Interventions without skill training were, however, only significant among indicated interventions (0.25, CI: 0.01 to 0.49, p = 0.042). Three of the 22 universal interventions, and eight of the 26 indicated interventions, assessed outcomes at follow-up (ranging between 13 to 52 weeks, and 2 to 26 weeks, respectively). Both universal and indicated interventions sustained significant positive effects on mental health outcomes at follow up (0.30, CI: 0.06 to 0.54, p = 0.015; 0.49, CI: 0.31 to 0.67, p<0.001, respectively).

Farrer et al. [50] systematically reviewed evidence on the effectiveness of technology-based interventions for mental health outcomes in tertiary students (rated as moderate methodological quality). They included both randomized controlled trials and randomized trials (equivalence trials). In interventions targeting both depression and anxiety, they found that technology-based CBT was effective in reducing anxiety and depression, although to a lesser degree than traditional therapy with human contact.

#### Other evidence

Conley et al. [51] conducted a meta-analysis of evidence (rated as moderate methodological quality) on the impacts of indicated prevention programmes for various forms of early-identified mental health problems such as sub-threshold depression and anxiety symptoms. Although they report significant effects, they provided insufficient information on the type of interventions to be categorised.

Rith-Najarian et al. [52] conducted a systematic review of evidence (rated as moderate methodological quality) on the effectiveness of preventative interventions in reducing depression, anxiety, and stress in university students. Rith-Najarian and colleagues found that prevention programmes reduced symptoms. The average effect sizes for preventative programmes were moderate (g = 0.65, 95% CI 0.57 to 0.73) regardless of delivery format or prevention level. According to delivery format, the effect sizes were similar for group (g = 0.69, 95% CI 0.58 to 0.81), self-administered (g = 0.65, 95% CI 0.50 to 0.81), and online/computer-delivered (0.52, 95% CI 0.41 to 0.63). According to prevention level, effect sizes differed for universal (0.69, 95% CI 0.55 to 0.83), selective (0.73, 95% CI 0.59 to 0.87), and indicated (0.53, 95% CI 0.44 to 0.63).

## Discussion

This review of reviews identified a range of interventions for student mental health and wellbeing, including mindfulness-based interventions (MBIs), psychological interventions (e.g., cognitive-behavioural therapy; CBT), psychoeducation interventions, recreation programmes, relaxation interventions, and setting-based interventions (e.g., academic and curriculum-based strategies). There was evidence that MBIs, CBT, and interventions delivered via technology were effective when compared to a passive control. There is some evidence to suggest that the effects of CBT-related interventions are sustained over time. The effects of interventions delivered via technology were found to be higher for interventions that were based on CBT principles in one higher quality review. Although technology-based CBT was effective in reducing depression and anxiety, traditional therapy with human contact was found to be more effective.

Moving beyond CBT, recreation programmes were also found to be effective. In fact, while both CBT and MBIs were found to be effective, other interventions (i.e., art, exercise, and peer support) were found to be more effective in one higher quality review. The review-level evidence suggests that psychoeducation interventions are not as effective as other interventions such as MBIs, cognitive-behavioural interventions, relaxation interventions, and meditation. The effects of psychoeducation interventions do not appear to sustain over time.

The review of reviews only located single reviews of evidence on acceptance and commitment training interventions [38] and setting-based interventions such as developing curricula to support wellbeing [43]. Although these interventions were shown to be effective, it should be noted that some of these reviews only included a small number of studies with small sample sizes [e.g., 38], and their findings should be viewed with some caution.

### Limitations in the review of reviews

This is the first review of reviews to synthesise evidence on interventions to improve college and university students’ mental health and wellbeing. Despite every effort to gather the best evidence available, the review had several limitations. First, as our searches were limited to English language literature, we did not include evidence from studies reported in other languages. Identification and synthesis of evidence published in other languages is therefore desirable, although this would require sophisticated, technical, multilingual skills during study identification, appraisal and synthesis. Second, the searches were limited to a 21-year date range (1999 to 2020). Although this date range was deemed appropriate as we aimed to identify interventions that are most relevant to modern student populations and contexts, it should be noted that this review of review-level evidence reflects the time period before the global COVID-19 pandemic. Last, scarcity of high quality evidence syntheses on interventions to improve student mental health and wellbeing led to our decision to analyse data from all 27 reviews. This decision impacts on the quality of evidence synthesised. Despite limitations in the methodological strength of some evidence, the search identified a substantial group of higher methodological quality reviews and a large number of systematic reviews and meta-analyses. It should, therefore, be used to inform policies and practice alongside other considerations.

### Gaps and limitations in the body of evidence

Although there was a large body of evidence on specific interventions such as mindfulness and cognitive-behavioural interventions, review-level evidence was limited in relation to other interventions such as setting-based interventions and acceptance and commitment training. Therefore, further primary studies examining the efficacy of setting-based interventions and acceptance and commitment training for students are required. Also, as there was a notable gap in the existing body of review-level evidence on interventions for students attending colleges in UK settings, a systematic review should be conducted in this area to identify primary level studies.

There are several limitations in the body of evidence. First, a number of the included reviews did not specify country and setting of the underlying evidence. It is likely that a substantial portion of the evidence is from US institutions, as this is typical for most evidence on health and wellbeing interventions. Another important limitation was that the included reviews only reported findings on beneficial effects of interventions. The underlying primary studies may have only attempted to assess efficacy and not the potential broader impacts of interventions. This is an important omission in the primary literature or the reviews. Interventions aiming for beneficial outcomes can often lead to unintended, adverse impacts for some participants. Primary and secondary research (including reviews) should attempt to identify adverse impacts so they can be eliminated or ameliorated, in accordance with the ‘first do no harm’ principle. A further limitation was that many of the included reviews did not consider the distribution of impacts from interventions across different population subgroups such as socio-economic status, age, gender, disability, and sexuality. As it is entirely possible that some interventions may work better for some students than for others, an evidence base that is more nuanced in terms of individual differences and differential impacts could underpin the tailoring of interventions to suit particular student characteristics leading, in time, to more suitable and effective interventions associated with nuanced, evidence-based delivery strategies. In addition to this, some of the included studies were lacking in detail on the nature of control groups. Greater detail on the nature of control groups should be provided in future studies. Last, few studies examined duration of effects over time. Future studies should routinely assess the duration of effects over time.

### Implications

In light of the above, future primary and review-level research should carefully consider the distribution of impacts of interventions by population sub-groups, including socioeconomic, gender, ethnic, age, sexuality, and disability groups [53]. Intersectionalities between these population characteristics should also be considered. Cultural and faith backgrounds may also be important factors to consider. Future research should also explore latency and durability of effects overtime as some interventions, such as CBT, showed promise of effects sustained post intervention. This could include exploring further and longer pre and post intervention studies and studies exploring the impacts of top-up sessions. Moving beyond CBT, there are wider social determinant interventions which may be particularly important in this context such as debt or financial management, quality of student accommodation and housing, the competitive versus cooperative ethos of the learning environment, and sense of belonging to the student body and to the institution [54]. With the increasing prevalence of student mental health issues pointing to the influence of these wider determinants, it is clear that primary research in this area that takes note of the distribution of impacts is needed.

## Conclusions

The review-of-reviews located a large body of evidence on specific interventions such as mindfulness and cognitive-behavioural interventions. The evidence suggests that these interventions can effectively reduce the common mental health difficulties of students. Evidence on other interventions was, however, limited. For example, although some work has begun developing curricula to support wellbeing, review-level evidence on organisational and structural interventions was limited. Thus, it is not currently possible to determine and rank which interventions work best, where and for whom, as this would require a larger body of evidence on certain intervention types, and comparative studies or reviews. Most of the included reviews did not consider the distribution of the intervention impacts (inequalities) for population subgroups such as age, gender, ethnicity, and socio-economic status. Noting the gaps and limitations in the review-level evidence previously identified, universities should select interventions based on the best available evidence, taking into consideration: the methodological strength of the underlying evidence, and the evidence on effectiveness. A good quality primary evidence-base examining these areas needs to be developed and then systematically reviewed before confident conclusions can be drawn about what works best to sustain positive mental health and wellbeing in today’s diverse and growing post-secondary student population. The need for effective support in this area can only have grown following the global COVID-19 pandemic and the associated disruption to teaching, learning, and university and college life. Following the disruption to teaching and learning, together with other stressors placed on young people from the COVID-19 pandemic, there is an imperative need to support students’ mental health and wellbeing. Future research in this area should elucidate the unique challenges that COVID-19 has presented for students to inform and tailor interventions for this generation and future cohorts facing disruptions to their teaching and learning experience.

## Supporting information

S1 Checklist (DOCX)

S1 File Search strategy. (DOCX)

S2 File AMSTAR 2 tool. (DOCX)

S3 File List of excluded studies. (DOCX)

S4 File Protocol. (DOCX)
