# Supplementary material for: Supporting mental health and wellbeing of university and college students: A systematic review of review-level evidence of interventions
Source: PLoS One. 2022 Jul 29;17(7):e0266725. doi: 10.1371/journal.pone.0266725 (PMC9337666; doi:10.1371/journal.pone.0266725)
Supplement: S1 File — (DOCX) [file pone.0266725.s002.docx]

**Supplementary file 1**

**SMH systematic review of reviews**

Search strategy (database syntaxes)

**MEDLINE; MEDLINE In Process and Other Non-indexed Citations.** Ran via OVID

| **N^o^** | **Terms** |
| --- | --- |
| 1 | (university student* OR undergraduate student* OR postgraduate student* OR college student* OR tertiary student* OR higher education OR tertiary education).ti,ab. |
| 2 | (mental OR wellbeing OR well-being OR depress* OR anxi* OR stress* OR resilience OR wellness OR coping OR mindfulness OR cognitive OR behavioural OR mediation).ti,ab. |
| 3 | (review OR synthes* OR meta-analysis OR overview).ti,ab. |
| 4 | AND 1-3 |
| 5 | Limit 4 to English Language, Humans, 1999 to current |

**Social Science Citation Index.** Ran via Web of Science

| **N^o^** | **Terms** |
| --- | --- |
| 1 | TS=(“university student*” OR “undergraduate student*” OR “postgraduate student*” OR “college student*” OR “tertiary student*” OR “higher education” OR “tertiary education”) |
| 2 | TS=(mental OR wellbeing OR well-being OR depress* OR anxi* OR stress* OR resilience OR wellness OR coping OR mindfulness OR cognitive OR behavioural OR mediation) |
| 3 | TS=(review OR synthes* OR meta-analysis OR overview) |
| 4 | AND 1-3 |
| 5 | Limit 4 to English Language, Humans, and 1999 to current |

**PsycINFO.** Ran via EBSCOhost

| **N^o^** | **Terms** |
| --- | --- |
| 1 | ( TI ((“university student*” OR “undergraduate student*” OR “postgraduate student*” OR “college student*” OR “tertiary student*” OR “higher education” OR “tertiary education”)) ) OR ( AB ((“university student*” OR “undergraduate student*” OR “postgraduate student*” OR “college student*” OR “tertiary student*” OR “higher education” OR “tertiary education”)) ) |
| 2 | ( TI ((mental OR wellbeing OR well-being OR depress* OR anxi* OR stress* OR resilience OR wellness OR coping OR mindfulness OR cognitive OR behavioural OR mediation)) ) OR ( AB ((mental OR wellbeing OR well-being OR depress* OR anxi* OR stress* OR resilience OR wellness OR coping OR mindfulness OR cognitive OR behavioural OR mediation)) ) |
| 3 | ( TI ((review OR synthes* OR meta-analysis OR overview)) ) OR ( AB ((review OR synthes* OR meta-analysis OR overview)) ) |
| 4 | AND 1-3 |
| 5 | # 3 Limited to English Language, and 1999-2019 |

**CINAHL Plus.** Searched via EBSCOhost.

| **N^o^** | **Terms** |
| --- | --- |
| 1 | ( TI ((“university student*” OR “undergraduate student*” OR “postgraduate student*” OR “college student*” OR “tertiary student*” OR “higher education” OR “tertiary education”)) ) OR ( AB ((“university student*” OR “undergraduate student*” OR “postgraduate student*” OR “college student*” OR “tertiary student*” OR “higher education” OR “tertiary education”)) ) |
| 2 | ( TI ((mental OR wellbeing OR well-being OR depress* OR anxi* OR stress* OR resilience OR wellness OR coping OR mindfulness OR cognitive OR behavioural OR mediation)) ) OR ( AB ((mental OR wellbeing OR well-being OR depress* OR anxi* OR stress* OR resilience OR wellness OR coping OR mindfulness OR cognitive OR behavioural OR mediation)) ) |
| 3 | ( TI ((review OR synthes* OR meta-analysis OR overview)) ) OR ( AB ((review OR synthes* OR meta-analysis OR overview)) ) |
| 4 | AND 1-3 |
| 5 | # 3 Limited to English Language, and 1999-2019 |
