# Supplementary material for: Supporting mental health and wellbeing of university and college students: A systematic review of review-level evidence of interventions
Source: PLoS One. 2022 Jul 29;17(7):e0266725. doi: 10.1371/journal.pone.0266725 (PMC9337666; doi:10.1371/journal.pone.0266725)
Supplement: S3 File — (DOCX) [file pone.0266725.s004.docx]

**List of excluded studies**

Abbing, A., Ponstein, A., van Hooren, S., de Sonneville, L., Swaab, H., & Baars, E. (2018). The effectiveness of art therapy for anxiety in adults: A systematic review of randomised and non-randomised controlled trials. *PloS one*, *13*(12), e0208716.

Akinla, O., Hagan, P., & Atiomo, W. (2018). A systematic review of the literature describing the outcomes of near-peer mentoring programs for first year medical students. *BMC medical education*, *18*(1), 1-10.

Cuijpers, P., Ebert, D. D., Acarturk, C., Andersson, G., & Cristea, I. A. (2016). Personalized psychotherapy for adult depression: a meta-analytic review. *Behavior Therapy*, *47*(6), 966-980.

Dietrich, S. K., Francis-Jimenez, C. M., Knibbs, M. D., Umali, I. L., & Truglio-Londrigan, M. (2016). Effectiveness of sleep education programs to improve sleep hygiene and/or sleep quality in college students: a systematic review. *JBI Evidence Synthesis*, *14*(9), 108-134.

Friedrich, A., & Schlarb, A. A. (2018). Let's talk about sleep: a systematic review of psychological interventions to improve sleep in college students. *Journal of Sleep Research*, *27*(1), 4-22.

Garrido, S., Millington, C., Cheers, D., Boydell, K., Schubert, E., Meade, T., & Nguyen, Q. V. (2019). What works and what doesn’t work? A systematic review of digital mental health interventions for depression and anxiety in young people. *Frontiers in psychiatry*, *10*, 759.

|  |
| --- |

Ghilardi, A., Buizza, C., Carobbio, E. M., & Lusenti, R. (2017). Detecting and managing mental health issues within young adults. A systematic review on college counselling in Italy. *Clinical practice and epidemiology in mental health: CP & EMH*, *13*, 61.

González-Valero, G., Zurita-Ortega, F., Ubago-Jiménez, J. L., & Puertas-Molero, P. (2019). Use of meditation and cognitive behavioral therapies for the treatment of stress, depression and anxiety in students. A systematic review and meta-analysis. *International journal of environmental research and public health*, *16*(22), 4394.

Harrod, C. S., Goss, C. W., Stallones, L., & DiGuiseppi, C. (2014). Interventions for primary prevention of suicide in university and other post‐secondary educational settings. *Cochrane database of systematic reviews*, (10).

Huntley, C. D., Young, B., Temple, J., Longworth, M., Smith, C. T., Jha, V., & Fisher, P. L. (2019). The efficacy of interventions for test-anxious university students: A meta-analysis of randomized controlled trials. *Journal of anxiety disorders*, *63*, 36-50.

Johnson, K. F., & Kalkbrenner, M. T. (2017). The utilization of technological innovations to support college student mental health: Mobile health communication. *Journal of technology in human services*, *35*(4), 314-339.

Li, C., Yin, H., Zhao, J., Shang, B., Hu, M., Zhang, P., & Chen, L. (2018). Interventions to promote mental health in nursing students: A systematic review and meta‐analysis of randomized controlled trials. *Journal of advanced nursing*, *74*(12), 2727-2741.

Lo, K., Waterland, J., Todd, P., Gupta, T., Bearman, M., Hassed, C., & Keating, J. L. (2018). Group interventions to promote mental health in health professional education: a systematic review and meta-analysis of randomised controlled trials. *Advances in Health Sciences Education*, *23*(2), 413-447.

Milne, T., Creedy, D. K., & West, R. (2016). Integrated systematic review on educational strategies that promote academic success and resilience in undergraduate indigenous students. *Nurse education today*, *36*, 387-394.

O’Driscoll, M., Byrne, S., Mc Gillicuddy, A., Lambert, S., & Sahm, L. J. (2017). The effects of mindfulness-based interventions for health and social care undergraduate students–a systematic review of the literature. *Psychology, health & medicine*, *22*(7), 851-865.

Shapiro, S., Brown, K., & Astin, J. (2011). Toward the integration of meditation into higher education: A review of research evidence. *Teachers College Record*, *113*(3), 493-528.

Witt, K., Boland, A., Lamblin, M., McGorry, P. D., Veness, B., Cipriani, A., ... & Robinson, J. (2019). Effectiveness of universal programmes for the prevention of suicidal ideation, behaviour and mental ill health in medical students: a systematic review and meta-analysis. *Evidence-based mental health*, *22*(2), 84-90.
