# Supplementary material for: Supporting mental health and wellbeing of university and college students: A systematic review of review-level evidence of interventions
Source: PLoS One. 2022 Jul 29;17(7):e0266725. doi: 10.1371/journal.pone.0266725 (PMC9337666; doi:10.1371/journal.pone.0266725)
Supplement: S4 File — (DOCX) [file pone.0266725.s005.docx]

**Supplementary file 4**

**Protocol**

**What interventions improve college and university student’s mental health and wellbeing? A review of review-level evidence.**

**Review team**

Joanne Worsley, Andy Pennington, Rhiannon Corcoran, University of Liverpool, for the What Works Centre for Wellbeing - Communities of Place evidence programme.

**Contact**

Joanne Worsley ([jworsley@liverpool.ac.uk](mailto:jworsley@liverpool.ac.uk)).

Eleanor Rathbone Building,

University of Liverpool,

Liverpool,

L69 7ZA

**Funding**

The What Works Centre for Wellbeing Communities of Place evidence programme is funded by the Economic and Social Research Council (ESRC) and partners.

Contents

[BACKGROUND 3](#_Toc12558119)

[Aim 3](#_Toc12558120)

[Review questions 3](#_Toc12558121)

[Definitions of key concepts 3](#_Toc12558122)

[Outputs from the review 3](#_Toc12558123)

[METHODS 4](#_Toc12558124)

[Identification of evidence 4](#_Toc12558125)

[Data extraction 5](#_Toc12558126)

[Assessment of methodological quality 6](#_Toc12558127)

[Data synthesis 6](#_Toc12558128)

[Transferability assessment 6](#_Toc12558129)

[Review advisors 6](#_Toc12558130)

[Review timeline 7](#_Toc12558131)

[REFERENCES 8](#_Toc12558132)

[APPENDICES 10](#_Toc12558133)

[Appendix 1. Sample search strategy (MEDLINE) 10](#_Toc12558134)

Appendix 2 [Source: Adapted from Pennington et al., 2017 11](#_Toc12558135)

Appendix 3. Quality Assessment template…………………………………………………………………………………12

# BACKGROUND

Early adulthood is known to be a period of peak risk for the onset of mental health difficulties (Kessler et al., 2007). As just under 50% of 18-19 year olds apply for university in the UK (UCAS Analysis and Research, 2014), students represent a substantial proportion of individuals in this high risk period. Poor mental health and well-being of university students is a growing public concern (Brown, 2018), and there is evidence that the prevalence of mental health difficulties in this population is increasing (Storrie, Ahern, & Tickett, 2010). In fact, there has been a fivefold increase in the proportion of students who disclose a mental health condition to their university over the past ten years (Thorley, 2017), and there has also been a substantial increase in the number of students seeking support from university counselling services (Thorley, 2017). Despite this increase, the capacity of such services to offer 1-1 support to large numbers of students is limited (Brown, 2018). There is therefore a need to understand which interventions are effective in improving college and university students’ mental health and wellbeing.

At present, the research on intervention effectiveness has not been well synthesised. Thus, this piece of work will identify existing evidence reviews on the topic and gaps in the coverage or strength of current evidence. This will allow additional questions to be devised to be answered by a systematic review.

## Aim

We will establish the evidence base for student mental health and well-being by delivering a review of reviews of what works to improve university student mental health. The aim of this review of reviews is to identify which interventions improve college and university students’ mental health and wellbeing.

## Review questions

1. What is the review-level evidence about the impact of interventions to improve the general mental health and wellbeing of college and university students in high-income OECD countries?
2. What are the most effective interventions to improve the general mental health and wellbeing of college and university students?

## Definitions of key concepts

General mental health

There are two aspects of mental health: positive mental health and mental ill-health. Positive mental health, also referred to as mental well-being, covers both affect and psychological functioning, as well as hedonic and eudaimonic well-being (Ryan & Deci, 2001). Mental ill-health is a broad term used to refer to phenomena that are often labelled or diagnosed as mental health problems. Mental health problems affect the way in which individuals think, feel, and behave.

## Outputs from the review

1. A technical report on the review of reviews.
2. A summary document in accessible language highlighting key findings from the review of reviews.
3. A peer reviewed academic publication.

# METHODS

The review will not attempt to search exhaustively for all relevant information, rather it will draw on systematic approaches to identify the most relevant evidence at review-level only. This will be informed by PRISMA and PRISMA-Equity guidelines (Moher et al., 2009; Welch et al., 2013).

## Identification of evidence

The search has been developed by experienced systematic review and information specialists.

The aim of the search is to identify all types of review-level evidence on wellbeing-related impacts of general mental health and wellbeing interventions for college and university students.

Given the limitations in the indexing of studies on wellbeing/wellbeing inequalities in electronic academic databases, broad freetext searches of study titles and abstracts in ‘gold standard’ academic databases will be conducted. The following electronic databases will be searched from 1999 to the present: MEDLINE, MEDLINE In Process and other Non-Indexed Citations; PsycINFO; Social Science Citation Index; CINAHL Plus. An example of the (MEDLINE) search strategy (syntax) is in Appendix 1.

Reference lists of all eligible (included) reviews will be hand-searched in order to identify additional relevant reviews.

Searches will be limited to studies published in English language only, as resources are not available to design and implement multilingual searches across academic and grey literature sources, or to interpret results reported in other languages. Searches will be limited to a 20 year date range (1999 to 2019) to locate the most recent evidence. To identify evidence that most relevant and transferable to UK settings, the searches will be limited to evidence from high income OECD countries only. Manual screening will be used to exclude reviews covering interventions on non-high income OECD countries.

The review inclusion and exclusion criteria and summarised in Table 1.

**Table 1. Review inclusion and exclusion criteria**

|  | **Include** | **Exclude** |
| --- | --- | --- |
| **P**opulation, setting | Post-secondary students attending colleges of further education or universities. All age groups including mature students. | Students at other levels of education (e.g. secondary) and settings (e.g. schools). |
| **I**ntervention | Interventions to improve general mental health and wellbeing. | Interventions to address specific, pre-existing mental health conditions or difficulties (e.g., attention deficit hyperactivity disorder). |
| **C**omparison | All control or comparator groups, or no control or comparator groups. | n/a |
| **O**utcome | All mental health and wellbeing outcomes. | Non-health or wellbeing outcomes, e.g., educational performance outcomes. |
| **S**tudy design | Review-level empirical studies (including evidence from qualitative, quantitative and/or mixed-method studies). | Primary-level studies. |
| Publication characteristics | English language publications.  Publications between 1999 and (09-05) 2019. | Studies published in other languages.  Studies published outside the date range 1999 and (09-05) 2019. |

Titles and abstracts of publications will be independently (double) screened by two reviewers based on the inclusion and exclusion criteria outlined in Table 1. Full-text copies of relevant papers will then be obtained and assessed for inclusion using the same criteria. Any queries or disagreements will be resolved by discussion or by recourse to a third reviewer. The screening and inclusion/exclusion process will be managed within EPPI Reviewer 4 systematic review management software.

## Data extraction

Data from each included study will be extracted into pre-designed and piloted forms. Forms will be completed by one reviewer and checked for accuracy by another. A random selection will be considered independently by two people for 20% of the studies. Data to be extracted include study aims, study design, setting/country, type of intervention, comparator (if any), population, outcomes reported, main findings in relation to the review questions, limitations and conclusions specified by authors. A data extraction template is in Appendix 2.

## Assessment of methodological quality

In a review of reviews, the legitimacy of the conclusions drawn is based on the results from the reviews that are included, which in turn are based on the results from the primary studies included in the review. Two questions, therefore, need to be answered: Was the review undertaken appropriately, and was quality assessment of the primary studies included in the review undertaken? These questions (and sub-questions) will be addressed through the use of a version of AMSTAR tool (Shea, 2007). A quality assessment template is shown in Appendix 3.

## Data synthesis

Key findings from the reviews will be tabulated and narratively synthesised (Mays et al., 2005; Popay et al. 2003; Whitehead et al., 2014). Findings will be grouped by intervention category, with evidence from higher methodological quality studies (reviews) being reported first and in greater detail. Gaps and limitations in the reviews and the underlying body of evidence will be identified were possible.

## Transferability assessment

Interventions that were designed, implemented and evaluated in other countries and settings may not always map well to other populations and settings (Bagnall et al., 2016; South et al., 2010).

## Review advisors

Dr Paul Litchfield, What Works Centre for Wellbeing

Nancy Hay, What Works Centre for Wellbeing

Silvia Brunetti, What Works Centre for Wellbeing

Gedminte Mikulenaite/John De Pury, Universities UK

Olga Tregaskis, University of East Anglia

Rosie Tressler/Gareth Hughes, Student Minds

Steven McAuliffe, University of Birmingham

Nicola Byrom, KCL, Student Mental Health Research Network

Kate McAlister/Jessica Trahar, Office for Students, Head of Student Welfare & Safeguarding

# Review timeline

#### Review start date

01 April 2019

#### Anticipated completion date

31 October 2019

**References**

Bagnall A, South J, Trigwell J, Kinsella K, White J, Harden A (2016) Community engagement – approaches to improve health: map of the literature on current and emerging community engagement policy and practice in the UK. Centre for Health Promotion Research, Leeds Beckett University / Institute for Health and Human Development, University of East London.

Brown, J.S.L. (2018). Student mental health: some answers and more questions. Journal of Mental Health, 27(3), 193-196.

Kessler, R. C., Amminger, G. P., Aguilar-Gaxiola, S., Alonso, J., Lee, S., & Ustun, T. B. (2007). Age of onset of mental disorders: A review of recent literature. Current Opinion in Psychiatry, 20(4), 359-364.

Mays N, Pope C, Popay J (2005) Systematically reviewing qualitative and quantitative evidence to inform management and policy-making in the health field. JHSRP, 10(1), S1:6-S1:20.

Moher D, Liberati A, Tetzlaff J, Altman D, and The PRISMA Group (2009) Preferred Reporting Items for Systematic Reviews and Meta-Analyses: The PRISMA Statement. PLoS Medicine, 6(7), 1-6.

Pennington A, Pilkington G, Bache I, Watkins M, Bagnall A, South R, Corcoran R (2017) A scoping review of review-level evidence on co-production in local decision-making and its relationship to community wellbeing. University of Liverpool: Liverpool.

Popay J, Roberts H, Sowden A, Petticrew M, Arai L, Rodgers M, et al. (2006) Guidance on the Conduct of Narrative Synthesis in Systematic Reviews. A product from the ESRC Methods Programme. Lancaster: Institute for Health Research, Lancaster University.

Ryan, R. M., & Deci, E. L. (2001). On happiness and human potentials: A review of research on hedonic and eudaimonic well-being. Annual Review of Psychology, 52(1), 141-166.

Shea B, Grimshaw J, Wells G, Boers M, Anderson N, Hamel C, et al. (2007) Development of AMSTAR: a measurement tool to assess the methodological quality of systematic reviews. BMC Medical Research Methodology, 7: 10.

South J, Meah A, Bagnall A, Kinsella K, Branney P, White J, and Gamsu M (2010) People in Public Health - a study of approaches to develop and support people in public health roles. Report for the National Institute for Health Research Service Delivery and Organisation programme. Leeds: Leeds Metropolitan University.

Storrie, K., Ahern, K., & Tickett, A. (2010). A systematic review: Students with mental health problems – a growing phenomenon. International Journal of Nursing Practice, 18, 1-6.

Thorley, C. (2017). Not by degrees: Improving student mental health in the UK’s universities. London: Institute for Public Policy Research.

University Central Admission Service. (2015). End of cycle report 2015: UCASE analysis and research.

Welch V, Petticrew M, Tugwell P, Moher D, O'Neill J, Waters E, and White H (2013) PRISMA-Equity 2012 Extension: Reporting Guidelines for Systematic Reviews with a Focus on Health Equity. Revista Panamericana de Salud Pública, 34(1): 60-67.

Whitehead M, Orton L, Pennington A, Nayak S, Ring A, Petticrew M, et al. (2014) Is control in the living environment important for health and wellbeing, and what are the implications for public health interventions? Final Report. London: Public Health Research Consortium.

# APPENDICES

## Appendix 1. Sample search strategy (MEDLINE)

**MEDLINE, MEDLINE In Process and Other Non-indexed Citations.** Ran via OVID.

| **N^o^** | **Terms** |
| --- | --- |
| 1 | (university student* OR undergraduate student* OR postgraduate student* OR college student* OR tertiary student* OR higher education OR tertiary education).ti,ab. |
| 2 | (mental OR wellbeing OR well-being OR depress* OR anxi* OR stress* OR resilience OR wellness OR coping OR mindfulness OR cognitive OR behavioural OR mediation).ti,ab. |
| 3 | (review OR synthes* OR meta-analysis OR overview).ti,ab. |
| 4 | AND 1-3 |
| 5 | Limit 4 to English Language, Humans, 1999 to current |

**Appendix 2. Data extraction template**

| **Author and Year** |  |
| --- | --- |
| **Title** |  |
| **Research/review question/s** |  |
| **Review aim, review objectives** |  |
| **Review inclusion criteria** |  |
| **Databases searched, date range** |  |
| **Number of primary studies included** |  |
| **Qual, quant and/or mixed-method** |  |
| **Primary study designs** |  |
| **Population** |  |
| **Location, setting/s** |  |
| **Intervention type/s** |  |
| **Outcomes measured** |  |
| **Synthesis method** |  |
| **Findings** |  |
| **Conclusions** |  |
| **Limitations in the review identified by authors** |  |
| **Author identified gaps and limitations in the evidence base, recommendations for future research** |  |
| Source: Adapted from Pennington et al., 2017 | |

**Appendix 3. Quality Assessment template**

|  | **Yes** | **No** | **Can’t answer** | **Not applicable** |
| --- | --- | --- | --- | --- |
| **1. Was an 'a priori' design provided?** The research question and inclusion criteria should be established before the conduct of the review. |  |  |  |  |
| **2. Was there duplicate study selection and data extraction?** There should be at least two independent data extractors and a consensus procedure for disagreements should be in place. |  |  |  |  |
| **3. Was a comprehensive literature search performed?** At least two electronic sources should be searched. The report must include years and databases used (e.g., Central, EMBASE, and MEDLINE). Key words and/or MESH terms must be stated and where feasible the search strategy should be provided. All searches should be supplemented by consulting current contents, reviews, textbooks, specialized registers, or experts in the particular field of study, and by reviewing the references in the studies found. |  |  |  |  |
| **4. Was the status of publication (i.e. grey literature) used as an inclusion criterion?** The authors should state that they searched for reports regardless of their publication type. The authors should state whether or not they excluded any reports (from the systematic review), based on their publication status, language etc. |  |  |  |  |
| **5. Was a list of studies (included and excluded) provided?** A list of included and excluded studies should be provided. |  |  |  |  |
| **6. Were the characteristics of the included studies provided?** In an aggregated form such as a table, data from the original studies should be provided on the participants, interventions and outcomes. The ranges of characteristics in all the studies analyzed e.g., age, race, sex, relevant socioeconomic data, disease status, duration, severity, or other diseases should be reported. |  |  |  |  |
| **7. Was the scientific quality of the included studies assessed and documented?** 'A priori' methods of assessment should be provided (e.g., for effectiveness studies if the author(s) chose to include only randomized, double-blind, placebo controlled studies, or allocation concealment as inclusion criteria); for other types of studies alternative items will be relevant. |  |  |  |  |
| **8. Was the scientific quality of the included studies used appropriately in formulating conclusions?** The results of the methodological rigor and scientific quality should be considered in the analysis and the conclusions of the review, and explicitly stated in formulating recommendations. |  |  |  |  |
| **9. Were the methods used to combine the findings of studies appropriate?** For the pooled results, a test should be done to ensure the studies were combinable, to assess their homogeneity (i.e., Chi-squared test for homogeneity). If heterogeneity exists a random effects model should be used and/or the clinical appropriateness of combining should be taken into consideration (i.e., is it sensible to combine?). |  |  |  |  |
| **10. Was the likelihood of publication bias assessed?** An assessment of publication bias should include a combination of graphical aids (e.g., funnel plot, other available tests) and/or statistical tests (e.g., Egger regression test, Hedges-Olken). |  |  |  |  |
| **11. Was the conflict of interest included? Potential** sources of support should be clearly acknowledged in both the systematic review and the included studies. |  |  |  |  |
